# Protective *versus *pathogenic anti-CD4 immunity: insights from the study of natural resistance to HIV infection

**DOI:** 10.1186/1479-5876-7-101

**Published:** 2009-11-28

**Authors:** Samuele E Burastero, Mariangela Figini, Barbara Frigerio, Paolo Lusso, Luca Mollica, Lucia Lopalco

**Affiliations:** 1 Unit of Clinical and Molecular Allergy, Division of Immunology, Infectious Diseases and Transplants, San Raffaele Scientific Institute, 58, via Olgettina, Milan, 20132, Italy; 2 Unit of Molecular Therapies, Department of Experimental Oncology and Laboratories, Fondazione IRCCS National Institute of Tumor, 1, via Venezian, Milan, 20132, Italy; 3 Laboratory of Immunoregulation, National Institute of Allergy and Infectious Diseases, National Institute of Health, Bethesda, MD 20892, USA; 4 Biomolecular NMR Laboratory, Dulbecco Telethon Institute, San Raffaele Scientific Institute, 58, via Olgettina, Milan, 20132, Italy; 5 Unit of Immunobiology of HIV, Division of Immunology, Infectious Diseases and Transplants, San Raffaele Scientific Institute, 58, via Olgettina, Milan, 20132, Italy

## Abstract

HIV-1 exposure causes several dramatic unbalances in the immune system homeostasis. Here, we will focus on the paradox whereby CD4 specific autoimmune responses, which are expected to contribute to the catastrophic loss of most part of the T helper lymphocyte subset in infected patients, may display the characteristics of an unconventional protective immunity in individuals naturally resistant to HIV-1 infection. Reference to differences in fine epitope mapping of these two oppositely polarized outcomes will be presented, with particular reference to partially or totally CD4-gp120 complex-specific antibodies. The fine tuning of the anti-self immune response to the HIV-1 receptor may determine whether viral exposure will result in infection or, alternatively, protective immunity.

Along this line, an efficacious anti-HIV strategy can rely on the active (*i.e*., through immunization) or passive targeting of cryptic epitopes of the CD4-gp120 complex, including those harboured within the CD4 molecule. Such epitopes are expected to be safe from genetic drift and thus allow for broad spectrum of efficacy. Moreover, since these epitopes are not routinely exposed in uninfected individuals, they are expected to become targets of neutralizing antibodies or other specifically designed molecules only after viral exposure, with a predictable low impact in terms of potentially harmful anti-CD4 self-reactivity.

The *experimentum naturae *of naturally resistant individuals indicates a strategy to design innovative strategies to neutralize HIV-1 by acting on the sharp edge between harmful and protective self-reactivity.

## 1. The paradox of CD4 T cell depletion in HIV-1 infection

Immune abnormalities are common features of both HIV infection and autoimmune diseases. The depletion of the CD4 T lymphocytes is the hallmark of the progression of HIV infection and, in the absence of antiviral treatment, the main contributor to the development of opportunistic infections and ultimately to the death of the majority of infected patients.

CD4 T lymphocytes physiologically play a central role in orchestrating the whole immune response, including the humoral and the cellular arms of acquired immunity against pathogens. Thus, it could be theoretically expected that a profound inhibition of immune cell activation would go together with CD4 cell death in HIV-1 infected persons. In contrast, levels of immune activation, as assessed by proportions of CD38+ DR+ T cells and serum concentrations of β2 microglobulin are closely correlated with disease progression and actually appear more accurate disease predictors than CD4 cell counts or viral load. HIV-1 infection leads to sustained activation of many key components of the immune system even in the very early stages and this is likely a fundamental mechanism for the ultimate collapse of immunity [1]. On the other hand, the natural host of SIV infection, sooty mangabeys, do not experience immune activation despite high levels of viral replication and this condition is associated with the absence of disease [2]. Similarly, the majority of HIV-2-infected subjects who remain free from HIV-induced immune suppression show negligible immune activation, whereas immune activation in progressor subjects with HIV-2 is comparable to that seen in HIV-1 infection [3]. In this scenario, the immune response to self antigens has often been alleged to play a detrimental role, by acting as an effector mechanism which indeed could explain how the relatively limited numbers of CD4 T cells actually infected by HIV-1 could bring to the catastrophic loss of this cell type during disease progression.

In particular, autoimmunity could contribute to impair CD4 T cell functions in HIV-1 infected persons *via *reactivity to the CD4 molecule itself. Indeed, in a pilot study by Keiser *et al*. [4] and in our own experience (S. Burastero, personal observation) anti-CD4 antibodies were found 90 to 540 days before the appearance of antibodies to HIV-1 in exposed individuals, suggesting that they may play a detrimental role since the first stages of HIV-1 infection. A recent study mapped the earliest anti-gp120 binding antibody responses to include the third variable region (V3) and reported that antibodies specific for CD4-induced epitopes, the CD4 binding site, and the membrane proximal external region of gp41 were not identified among early anti-Env responses [5]. Moreover, Davis *et al*. reported that high-titre, broadly reactive V3-specific antibodies are among the first to be elicited during acute and early HIV-1 infection and following vaccination. However, these antibodies lacked neutralizing potency against primary HIV-1 viruses, which effectively shield V3 from antibody binding to the functional Env trimer [6]. In this context, dedicated parallel studies are needed to accurately define the timing of appearance of anti-CD4 antibodies, particularly to gp120-induced epitope, as compared to anti-Env antibodies

## 2. Mechanisms for breaking of tolerance following HIV-1 exposure

### 2a) Cell death and apoptosis

Several mechanisms were studied, which could support the development of autoimmunity in HIV-1 infected persons. Oswald-Ritchter *at al*. proposed a specific susceptibility of regulatory T cells to HIV-1 infection [7] whereas Rawson *et al*. [8] focused on the increased tendency of CD4 T lymphocytes from infected individuals to undergo activation-induced death or apoptosis and demonstrated the subsequent presentation of remarkable amounts of self-epitopes. This second mechanism was found capable to break tolerance and trigger cytotoxic T cell-mediated autoreactivity towards several autoantigens, such as myosin, vimentin and actin [8], promoting the formation of autoreactive CD8 T cells. Apoptosis is an ordered state of cell death in which the structural components of the cell are carefully disassembled by the activity of a unique set of proteolytic enzymes, notably members of the caspase family [8]. The self-proteins broken down by caspases in a multitude of apoptotic cells can also prime cytotoxic T lymphocytes (CTLs) through subsequent proteasomal digestion and cross-presentation. Thus, the massive death and destruction of lymphocytes in HIV-1 infection could break tolerance to self-peptides and permits the generation of autoreactive CTLs responding to the cleavage products of apoptotic cells.

### 2b) Immunodeficiency and autoimmune phenomena in lentiviral infection of non-human primates

Both SIV-infected Rhesus macaques and Sooty Mangabeys, species from Africa are naturally infected with SIV, yet they do not display any detectable signs of immune deficiency or autoimmunity. On this basis, they have been used as models to explore the possible mechanism underlying the generation of autoimmune phenomena in HIV-1 infected humans [9]. In one crucial observation based on *ex vivo *CD4 T cell depletion, the availability of activated CD4+ T cells, rather than immune control of SIV replication, appeared the main determinant of viral load during natural SIV infection of Sooty Mangabeys [10]. Moreover, in blood and tissues of rhesus macaques inoculated with derivatives of the pathogenic SIVsmE543-3 or SIVmac239, phenotypic analysis of CD4(+) T cells demonstrated two patterns of depletion, primarily affecting either naïve or memory CD4(+) T cells [11], respectively. In this experimental setting, progressive decline of total CD4(+) T cells was observed only in those macaques with naïve CD4(+) T cell depletion and the level of autoreactive antibodies correlated with the extent of naive CD4(+) T cell depletion. These results suggest an important role of autoreactive antibodies and of naïve T cells in the CD4(+) T cell decline observed during progression to AIDS [11].

### 2c) Cryptic epitopes and inter-molecular help can generate anti-CD4 auto-reactiviy

An autoimmune cytotoxic T-cell response to the CD4 molecule was described in HIV-1 positive patients [12,13]. The unveiling of cryptic epitopes following internalization of CD4 in complex with gp120 was proposed to explain the pathogenesis of this phenomenon [14,15]. A further *in vivo *proof of principle of the importance of this mechanism was provided by Abulafia-Laid *et al*. [16], who showed the efficacy of T-cell vaccination against anti-CD4 autoimmunity in a small sample of HIV-infected patients. Intracellular interactions of newly synthesized CD4 molecules with various HIV proteins may be the basis for the generation of various self-epitopes, which in the absence of HIV are ignored due to tolerance mechanisms. In fact, the formation of Env (gp160)-CD4 complexes in the ER can lead to their retention *via *binding to Vpu, which re-direct them to degradation [17-20]. Similarly, Nef interaction with the cytoplasmic tail of membrane CD4 was reported to prompt its transport to degradation organelles [21]. Thus, autoimmunity to CD4 in HIV-1 infected patients is supported by several mechanisms associated with the generation of cryptic epitopes and to the activation of T cells not previously deleted by central tolerance during the maturation of the T cell repertoire.

An alternatively, not mutually exclusive hypothesis for the generation of anti-CD4 antibodies is the so-called "inter-molecular help" phenomenon. This mechanism implies that gp120-specific T cells can help antibody production by CD4-specific B cells, which could recognize B-cell epitopes on a gp120-CD4 complex [22]. Although the *in vivo *relevance of this specific occurrence has never been established, it should to be considered as a reasonable possibility, reminiscent of the more general occurrence of redirected antigen-presentation, which follows presentation of antigens complexed with antibodies with different fine specificities [23,24]. However, this mechanism would imply that gp120-specific immunity would necessarily precede CD4-autoimmunity, whereas there is evidence in contrast to this scenario [4].

## 3) Anti-CD4 antibodies in clinical practice: beyond immune suppression

As expected from basic immunology notions, anti-CD4 antibodies have long been proposed and used as immune suppressors, *e.g*., in clinical trials for the treatment of human autoimmune diseases [25]. In early studies, anti-CD4 mAbs were found capable to induce either cell depletion [26] or functional inactivation of T cells [27,28], although activation of T-cell functions was also reported[29]. These divergent effects may explain the inconsistency in the clinical efficacy of different anti-CD4 mAbs particularly in the treatment of rheumatoid arthritis, namely a promising initial efficacy in open anti-CD4 trials [30,31], subsequent discouraging double-blind clinical trials (reviewed in [32]), and, finally, a revitalization of the anti-CD4 treatment notion with new, humanized anti-CD4 mAbs [33]. Indeed, the usage of this approach has been hampered by the complexity of its effects on the immune system. For instance, it has long been known that anti-CD4 monoclonals are immune suppressive or tolerogenic depending on the circumstances of their administration [34-36]. Moreover, it is generally recognized that non-depleting monoclonal may be relatively more effective in tolerance induction, for instance in the treatment of rheumatoid arthritis [30], psoriasis [37], systemic lupus erythematosus [38] and multiple sclerosis [39], although only inconclusive and temporary symptom relief was achieved in open studies. The fine epitope specificity of anti-CD4 antibodies may play a role in this context, since in rat adjuvant arthritis the developmental pattern of arthritis differed substantially between three distinct monoclonals, two of them preventing, the third one accelerating the development of the disease [40]. The effect of each reagent on the signaling activated by CD4 *via *the p56_lck _interacting cytoplasmic tail is supposedly implicated in these differences.

In this context, the usage of human derivatives of mouse monoclonals allowed not only to reduce the generation of xenogeneic reactivity of rodent monoclonals, but also to modulate induced effector mechanisms. In engineered derivatives, the isotype used (*e.g*., IgG1 versus IgG4) has implication on complement fixation capability and on the binding to Fc receptors bearing cells, whereas variation in the number of binding sites (*e.g*., single chain constructs, Fc fragments, *etc*.) implies modification of functional effects of the original reagent. Recently, a fully human anti-CD4 monoclonal antibody (HuMax-CD4) was tested in a multicenter, double blind, placebo-controlled, randomized clinical trial on 85 moderate to severe psoriasis patients, showing decreases in the psoriasis skin score, although this failed to reach statistical significance [41].

Further complexity to be considered when using *in vivo *CD4-interacting reagents derives from the fact that two sets of NFAT binding sites were identified in the HIV-1 long terminal repeat (LTR) promoter, and CD4 engagement can result on the p56_lck _kinase dependent activation of both cellular transcription factors and HIV-1 LTR [42]. Thus, a signaling trigger *via *CD4 can activate both the endogenous and the retroviral NFAT family of transcription factors, simultaneously inducing both T cell activation and increased transcription of the viral genome [43]. This phenomenon was implicated to explain the observation that HIV-1-positive transplant recipients reduced viral burden during treatment with cyclosporin A (CsA) [44], a potent inhibitor of these transcription factors. Moreover, CD4 dimerization occurs when CD4 membrane cell density exceeds 10^5 ^per cells, involves D4-D4 domain interactions and *per se *triggers auto-phosphorylation and T cell activation [45].

Thus, the effect of anti-CD4 in human therapy is far from being a straightforward immune suppression and is influenced by so different factors as epitope specificity, isotype and number of binding sites.

Recently, one anti-CD4 antibody (ibalizumab) which does not induce any relevant immune suppressive effect *in vitro *or *in vivo *was tested in phase II clinical trials, in the form of human IgG4 derivative, and appeared a promising tool to block HIV-1 infection without inducing any immunologically relevant side-effect [46,47]. This molecule recognizes a CD4 D2 epitope and does not significantly interferes with HIV-1 docking on the cell membrane. The anti-viral activity of ibalizumab is explained as a consequence of the interference on conformational changes taking place on the cellular HIV-1 receptor at the post-binding level [48].

## 4) Antibodies to the CD4-gp120 complex

Following CD4-gp120 interaction, a sequence of pre-ordered conformational changes takes place on both moieties of the complex. These conformational modifications are non-optional events, which allow gp120 interaction with coreceptor and prompt membrane fusion and viral entry into the cells. From the immune system perspective, this conformational flexibility generates a series of transitorily expressed antigenic determinants, which re-design the epitopic make up of interacting moieties.

Along this line, a complementary and reciprocal observation came from a recent study focused on alterations in the antigenicity and immunogenicity of gp120 when complexed with monoclonal antibodies specific for the CD4 binding site of gp120 [49]. Results indicated that these antibodies enhanced production of anti-gp120 antibodies directed particularly against the V3 region[49]. These data further support the notion that immune responses can be induced specifically against unique epitopes created upon the interactions of CD4 with gp120, with monoclonal antibodies, or other ligands.

The binding of gp120 to CD4 involves a well-defined site within the first Ig-like domain of CD4 (CD4 D1) [50]. The Phe43 CD4 residue plays a non-optional role in this crucial interaction [51] by docking into a conserved hydrophobic pocket, a discontinuous region at the interface between the inner and the outer domain of gp120 [52]. On the other hand, the lateral face of the D1 CD4 domain is implicated in MHC-class II interaction, which physiologically provides an activation signal and plays a key role in the physiological and pathological T lymphocyte functions [53].

Notably, the OKT4A monoclonal antibody specifically binds to the gp120 binding site of CD4, and displays, as expected, a remarkable anti-HIV activity *in vitro*. However, this reagent is also extremely immune suppressive due to interference on the physiological CD4 function. CD4 induced (CD4i) are those epitopes, which are exposed on the gp120 molecule after binding to the cellular receptor. All known CD4i antibodies recognize a common, conserved gp120 element overlapping the binding site for the CCR5 chemokine receptor [54]. Recently, we characterized a gp120 neutralization epitope, recognized by the D19 murine monoclonal antibody, which is differentially accessible in the native HIV-1 Env according to its coreceptor specificity [55]. In CCR5-restricted (R5) isolates, the D19 epitope was invariably cryptic, although it could be exposed by the addition of soluble CD4; epitope masking was dependent on the native oligomeric structure of Env, since it was not observed with the corresponding monomeric gp120 molecules. By contrast, in CXCR4-using strains, the D19 epitope was constitutively accessible. In accordance with these results, R5 isolates were resistant to neutralization by D19, becoming sensitive only upon addition of sCD4, whereas CXCR4-using isolates were neutralized regardless of the presence of sCD4 [55]. Taken together, these observations can be deciphered in evolutionary term by saying that CD4-induced changes in gp120 conformation are functionally crucial for HIV-1 entry, and illustrates a viral strategy for sequestering the chemokine receptor-binding region of gp120 away from the attacks of the humoral immune response [56].

In a reciprocal fashion, similar observations can be applied to the CD4 receptor. Complex specific epitopes on the CD4 moiety have been identified with partially or totally complex-specific monoclonals antibodies, which do not interfere with the CD4-Env complex formation, such as CG10 [57] and antibody 55 [58], both mapping to the second Ig-like CD4 domain. We recently generated an anti-D2 CD4 monoclonal antibody (DB-81) [59,60] not interfering with gp120 binding and with a binding affinity around 700 times higher for CD4 complexed to gp120, as compared to CD4 (Burastero S, Lusso P, *et al*., in preparation). Notably, CG10 is weakly interfering with membrane fusion and HIV replication [57], whereas antibody 55 [58] and DB-81 react with both membrane-bound and solid-phase coated recombinant CD4 and display a broad spectrum of neutralization, suggesting that little differences in the fine specificity may imply relevant impact on the capability to interfere with the chain of events which follows viral docking on the cell membrane.

**Figure 1:**
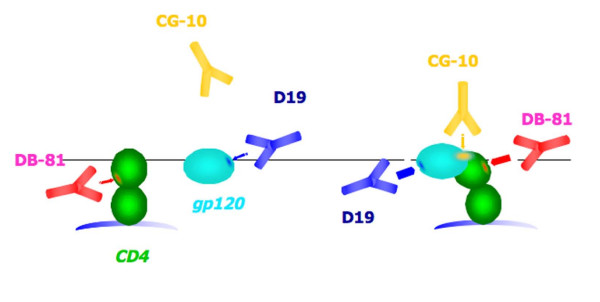
**Schematic representation of the interaction between CD4 and gp120, with reference to the formation of new epitopes**. The indicated monoclonal antibodies are either exclusively (CG10) or preferentially (DB-81) binding to CD4 complexed to gp120 (right part of the figure), as compared to CD4 only (left part of the figure). Similarly, the anti-gp120 D19 monoclonal antibody is represented, which binds with higher affinity to gp120 complexed to CD4, as compared to (R5-coreceptor restricted) gp120 only. The affinities of the antigen-antibody interactions are proportional to the thickness of the arrow pointing to the epitope.

A visual representation of conformation-specific epitopes generated following CD4-gp120 interaction is depicted in figure 1, where the binding of some of the above quoted monoclonals is represented. In Table 1 the basic mechanisms of protection by representative CD4 binding monoclonals are listed.

**Table 1:** MECHANISMS OF HIV-PROTECTION BY ANTI-CD4 ANTIBODIES

*1) interference with gp120 binding*			
*Antibody*	*Binding site*	*Binding to CD4-gp120 complex*	*Characteristics*

OKT4a[76]	First CD4 domain	Does not occur due to epitope masking	Difficult to generate *in vivo* Immune suppressive

			
***2) interference with the sequence of conformational modifications subsequent to gp120 binding and permissive to coreceptor binding and membrane fusion***			

*Antibody*	*Binding site*	*Binding to CD4-gp120 complex*	*Characteristics*

Ibalizumab[46]	Second CD4 domain	Equivalent binding to free and complexed CD4	Non immune suppressive

DB-81[59,60]	Second CD4 domain	Increased binding to complexed CD4	a) Non immune suppressive; b) Fine specificity shared by ESN individuals

## 5) Fine specificity of anti-CD4 antibodies in HIV-1 exposed individuals with different susceptibility to HIV infection

It has long been known that autoimmune responses towards CD4 detected in HIV-1 infected individuals are produced (by the breaking of immune tolerance) which seem to discriminate soluble *versus *cell associated CD4 antigens.

In fact, it was consistently reported that these antibodies bind to solid-phase recombinant CD4, but fail to recognize CD4 expressed on the surface of CD4+ lymphocytes or cell lines [61,62](Burastero, personal observations). These antibodies are mainly directed against a region of the viral receptor distinct from the virus-binding domain [63] and preferentially recognize epitopes masked by the physiological dimerization of CD4 on the cell membrane. This observation suggests that they are derived from such an extensive processing of the self antigen that hidden epitopes "emerged" on antigen presenting cells and were exposed efficiently enough to become the target of humoral immunity.

Consistently with these findings, extensive epitope scanning mapped CD4-specific T cells in HIV-1 positive individuals to any of the four CD4 domains [64]. In contrast, the little proportion of CD4-reacting IgG from healthy individuals are specific for epitopes of extracellular CD4 domains (*ibid*.).

Recently, Denisova *et al*. [45] reported that immunization of hu-CD4 C57Black/6J mice with HIV-1 gp120(451) complexed with its receptor protein produced, in the tolerogenic hu-CD4 transgenic background used to mimic the human situation, two anti-CD4 monoclonal antibodies, designated T6 and T9, mapping to the D3-D4 domains and recognizing soluble but not membrane associated CD4. These antibodies were capable to compete with anti-CD4 antibodies detected in HIV-1 infected people.

In contrast to this situation, a surprise came from individuals with natural resistance to HIV-1 infection. Far from being immunologically non-reactive, these HIV-exposed, uninfected subjects (ESN) display several unconventional autoimmune traits, including the distinctive reactivity towards the CD4 molecule [65]. An inter-molecular help mechanism could explain the breaking of tolerance and the switch to the IgG isotype of these antibodies [66]. Also newborn babies from seropositive mothers were found to display this autoimmune trait, which disappeared following spontaneous viral clearance [67]. These antibodies are likely part of a more general anti-cell immunity, including specificities to CCR5, the HIV- coreceptor [68].

Notably, anti-CD4 antibodies in ESN subjects bind to both membrane and soluble CD4 and have syncytium inhibiting activity [65]. The distinct fine specificity of anti-CD4 antibodies in exposed uninfected, naturally resistant, *versus *HIV-1 infected people was later confirmed in a larger cohort of individuals, where a clear-cut prevalence of complex-specific antibodies in the former was reported, suggesting a possible protective role [68]. This notion was also supported by preliminary observations with anti-CD4 sera form long-term non-progressor patients [69].

Thus, anti-CD4 antibodies in ESN subjects are one among several signs of unconventional immunity, which were described in HIV-1 resistant individuals [70]. We speculate that specificity to the first two domains of membrane CD4, with particular reference to strictly conformation-dependent epitopes, and including those, which are preferentially expressed after gp120 binding may be associated with a non-harmful and potentially protective humoral anti-HIV-1 autoimmune response.

Further studies are needed to characterize anti-CD4 antibodies fine specificities in healthy subjects, with or without HIV- exposure, and to determine their HIV-1 inhibitory capability.

**Figure 2:**
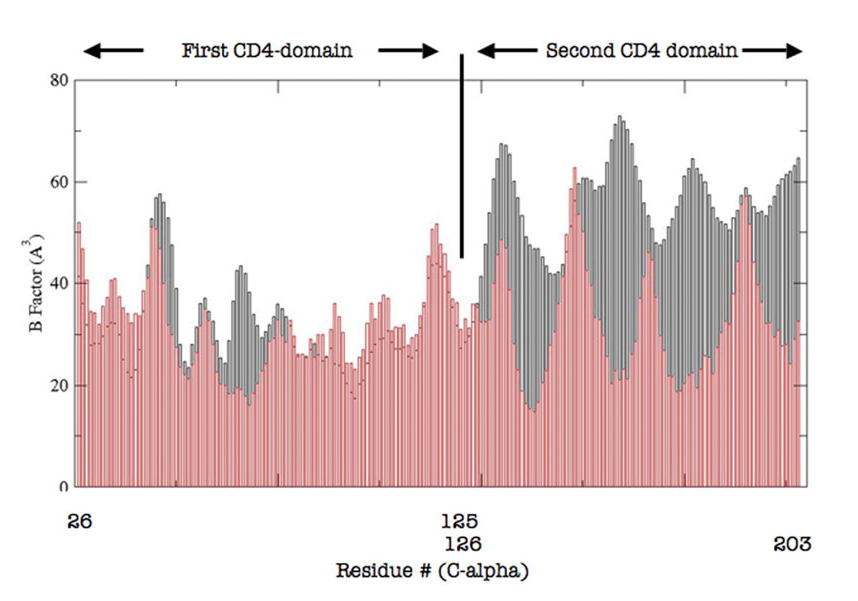
**B factors (as a measure of local backbone mobility, on the *y*-axys) of C-alpha atoms for the free (gray) and the gp120-complexed (red) CD4 protein (C-alpha residue numbering is on the x-axis, according to UniProtKB/Swiss-Prot **P01730).The first Ig-like V-type (residues 26 -- 125) and the second Ig-like C2-type 1 (residues 126 -- 203) were included in this analysis. Data were calculated from PDB files 3CD4 and 2NXY for free and complexed CD4, respectively. The third and forth domains were not considered due to the expected influence on B factors of these portions of the molecule by physiological CD4 dimerization.

**Figure 3:**
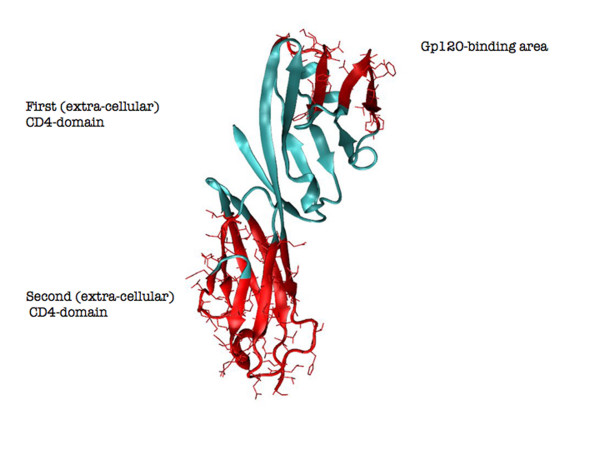
**Regions of CD4 structure (within the first and the second CD4 domain) that display (in red) the greatest changes in C-alpha B factor between the free form and the one complexed with gp120**. The C-alpha B-factor was calculated as a measure of local backbone flexibility.

Molecular structure analysis of free versus unbound CD4 may be helpful in shedding light on the above reported observations. Here, the two structures backbones were aligned and they resulted to be almost completely overlapping (Root Mean Square Distance < 0.7 Å). C-alpha atoms B-factors were then extracted from the PDB files of the compared structures (accession numbers 3CD4 and 2NXY for CD4 and CD4-gp120 complex, respectively) as a measure of local backbone mobility [71](Figure 2). The first CD4 domain did not display significant variations of local backbone mobility with the expected exception of the region in close contact with the surface of gp120. In contrast, the second domain displayed large variations which mapped the majority of the structure (Figure 3). This result suggests that the D2 CD4 domain significantly reduces its local flexibility, despite the fact that it is not directly involved in binding, whereas the D1 CD4 domain remains virtually unaltered in its local mobility. Thus, it appears that the conformation of the membrane molecule serving as viral receptor has a defined degree of flexibility of solvent-exposed determinants, which is decreased following ligand binding. This decrease occurs not only, as expected, in the direct proximity of the binding site, but also in extended portions of the second CD4 domain.

**Figure 4:**
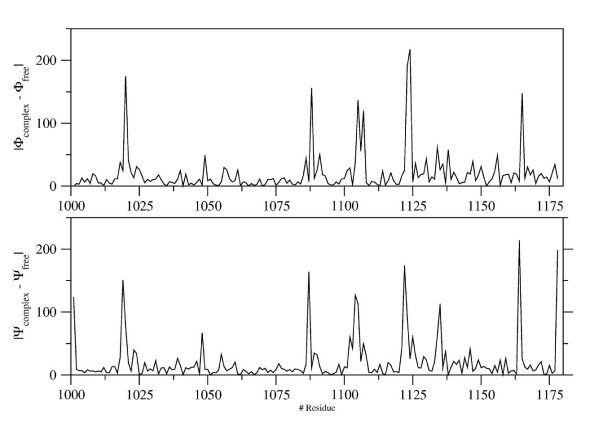
**Local differences in the conformation of CD4 in the gp120-bound *versus *free state**. Absolute variations of dihedral backbone angles Φ (upper panel) ad Ψ (lower panel) between bound and free CD4 structure are plotted on the *y*-axis against the single residues (on the *x*-axis) whose local geometry is influenced by the interaction between the two moieties.

In order to further highlight these local differences, in figure 4 the variations of dihedral angles (Φ and Ψ) between the bound and the free state are plotted against the single residues whose local geometry is influenced by the binding of the two moieties.

The pheomenon of complex-dependent conformational variations may be exploited to augment the chances of inhibiting viral entry by increasing the opportunity for binding to occur by strictly conformational antibodies, or derivatives thereof, specific to such protruding "stiffer" epitopes. Since such a locally rigid antigenic make up is by definition transient, and the corresponding set of epitope is limited, it may be in principle associated with an overall lower immunogenicity. However, available data on anti-CD4 antibodies in ESN demonstrate that a proportion of individuals can indeed spontaneously produce antibodies with these fine specificities. These may pre-exist as the results of previous exposure to different (non HIV-related) antigenic stimuli, they may be natural antibodies with relatively low affinilty, and/or may be subjected to affinity maturation following HIV-1 exposure. The propensity to assume a different conformation as compared to the native one was also found associated to increased immunogenicity and antibody affinity in immunization experiments performed with CCR5-ECL-1 loop after alanine substitution [72]. In that context, this finding led us to hypothesize that flexibility of some conformed regions can change their status upon antigenic stimuli and prove helpful in enhancing immunogenicity and eliciting high affinity HIV protective antibodies.

## Conclusion

Individuals naturally resistant to HIV-1 infection represent an experiment of nature whose study has potential implication for the design of alternative immunological therapies of HIV-1 infection. Anti-CD4 antibodies are not subjected to the immune evasion, which characterize Env-specific immunity, nor to the generation of resistance, which impairs the efficacy of antiretroviral therapy with non-entry inhibitors. Thus, the possibility to elicit non-immune suppressive, protective anti-CD4 immune responses or, alternatively, to use monoclonal antibodies or derivatives thereof, which will reproduce this activity may dramatically improve therapeutic options for HIV-1 treatment in the next few years.

A long-standing effort has been attempted to target conformation-specific epitopes, as a strategy to overcome the failure of conventional vaccination approaches to prevent HIV-1 infection [73-75]. The data we review here suggest that the fine characterization of crucial epitopes recognized by antibodies from ESN subjects will allow to increase the chances to successfully implement this strategy

## List of abbreviations

ESN: Exposed Sero-Negative.

## Competing interests

The authors declare that they have no competing interests.

## Authors' contributions

SB and LL coordinated several studies on ESN subjects, aimed to characterize defined aspects of conventional and non-conventional immunity against HIV and the HIV receptor/co-receptor. PL and SB coordinated studies aimed to reproduce in a mouse-based animal model the generation of a humoral immunity mimicking some specific features of that observed in ESN individuals. LM performed structural biology studies to characterize epitopes recognized on the CD4 molecule by antibodies from ESN individuals and by mouse immunized with membrane-bound CD4-gp120 complex. MF and BF characterized the fine specificity and the binding characteristics (K_on_, K_off_, affinity) of antibodies from ESN individuals and from mice immunized with membrane-bound CD4-gp120 complex. Moreover, MF and BF generated several human derivatives of single mouse monoclonals recapitulating these characteristics. All authors read and approved the final manuscript.
